# Alternative Lengthening of Telomeres: The Need for *ATRX* Mutations Is Lineage-Dependent

**DOI:** 10.3390/ijms26146765

**Published:** 2025-07-15

**Authors:** Ion Udroiu, Antonella Sgura

**Affiliations:** Department of Science, Roma Tre University, Viale G. Marconi 446, 00146 Rome, Italy; antonella.sgura@uniroma3.it

**Keywords:** alpha thalassemia, carcinoma, histology, mutation, neuroblastoma, sarcoma, telomere

## Abstract

During carcinogenesis, cells must acquire a telomere maintenance mechanism in order to avoid telomere shortening-induced replicative senescence. While most tumors activate telomerase, a minority of them employ a recombinational mechanism called Alternative Lengthening of Telomeres (ALT). One of the most investigated features is the association between ALT and *ATRX* mutations, since this has been shown to be the gene with the highest rate of mutations among ALT tumors. However, most of these studies, and in particular, mechanistic studies in vitro, have been carried out on mesenchymal tumors (sarcomas). In the present study, using genomic and expression data from the DepMap portal, we identified several non-mesenchymal ALT cell lines, and we compared the incidence of *ATRX* and other gene mutations between ALT cell lines of different origins (mesenchymal, neural, epithelial, hematopoietic). We confirmed that *ATRX* is frequently mutated in mesenchymal and neural ALT cell lines but not in epithelial ones. Our results showed that mutations of ATRX or other proteins involved in the maintenance of telomere integrity are needed for ALT activation in all cell types, and ATRX is preferentially mutated in mesenchymal ALT cells. Besides a more precise interpretation of the role of ATRX loss in ALT establishment, we proposed a model in which mutation of this gene impairs differentiation in mesenchymal and neural cells (but not in epithelial ones). Therefore, we explained the high incidence of *ATRX* mutations in mesenchymal and neural tumors with the fact that they both trigger ALT and impair differentiation, thus promoting two steps at once in the process of carcinogenesis.

## 1. Introduction

Cells with unlimited proliferation are characterized by the ability to avoid telomere shortening. This is achieved either by activation of telomerase or by a homologous recombination (HR)-based mechanism called ALT (Alternative Lengthening of Telomeres). These telomere maintenance mechanisms (TMMs) have been identified by many researchers as tumor-specific targets for possible therapies.

Many phenomena that lead to telomerase activation have been discovered, i.e., *TERT* (the gene encoding for the catalytic subunit of telomerase) amplification, mutations of the *TERT* promoter, and mutations/amplifications of *TERT*-activating oncogenes [1].

Concerning ALT, knowledge of its mechanisms has made progress in the last two decades, whereas the understanding of its activation remains poor [2]. One of the most investigated features is the association between ALT and ATRX mutations [3,4]. ATRX is a helicase and chromatin remodeler, a member of the SWI/SNF family, which, forming a complex with DAXX, deposits the histone variant H3.3 on telomeric, pericentromeric, and other heterochromatic regions [5]. It also plays a role in the establishment of Senescence-Associated Heterochromatic Foci [6] and is essential for replication stress tolerance and restart at stalled replication forks [7]. A hypothesis shared by many researchers is that in the absence of ATRX, H3 histone is not deposited at telomeres, which thus turn from heterochromatic to euchromatic, creating an environment permissive for ALT [8].

In vitro studies aimed at activating ALT by ATRX silencing gave conflicting results [9]. Nonetheless, *ATRX* remains the gene with the highest rate of mutations among ALT tumors. Moreover, Lovejoy et al. [10] studied many ALT cell lines, investigating both the presence of ALT markers and ATRX alterations. The results showed that almost all ALT cell lines, i.e., 16/22 (73%), were ATRX deficient. However, the composition of this cell line panel was heavily biased by its mesenchymal origin, as three cell lines were osteosarcomas and 17 were transformed fibroblasts (many of which were subclones from the same individual). Presenting the data in another way, it can be seen that 67% of osteosarcoma cell lines (2/3) and 82% of fibroblast cell lines (14/17) were ATRX deficient, whereas a lung cancer cell line (SKLU-1) and a mesothelial cell line (MeT-4A) were both ATRX proficient. From these data (although few), it could be argued that the incidence of ATRX loss is high among ALT cells of mesenchymal origin but low among those of epithelial origin. Moreover, another difference related to the histological types of tumors is that the incidence of ALT has been reported as far higher among (mesenchymal) sarcomas than in (epithelial) carcinomas [11,12].

The aim of this paper was to compare the incidence of ATRX and other gene mutations between ALT cell lines of different origins (mesenchymal, neural, epithelial, hematopoietic).

## 2. Results

### 2.1. Identification of ALT Cell Lines

Among the 1854 CCLE cell lines in the DepMap portal [13], using literature data, 16 cell lines were characterized as ALT, 206 as telomerase-positive and three as tumor cells without TMM (Table S1 in Supplementary Material). The unequivocal criterion is the presence of telomerase activity for telomerase-positive cell lines, while for ALT cells it is the absence of telomerase activity and the presence of ALT markers (long and heterogeneous telomeres, C-circles, ALT-associated PML Bodies). The other cell lines for which TMM characterization is absent in the literature were classified as “unknown”.

**Figure 1:**
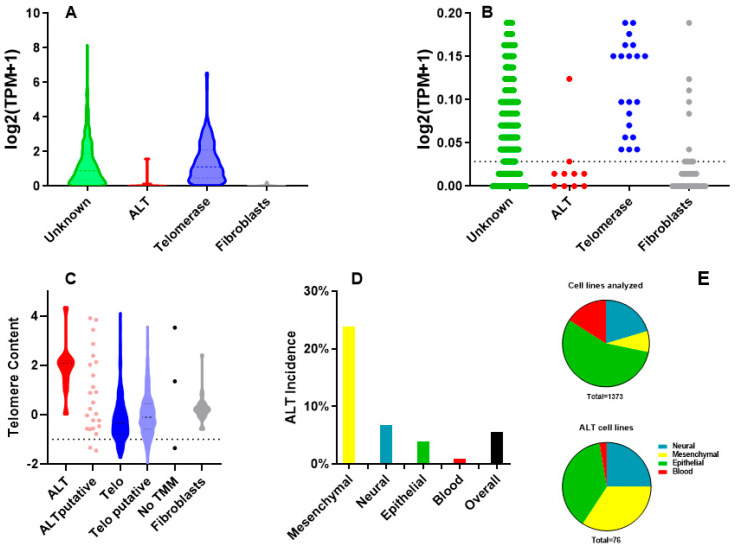
Identification of ALT cell lines. (**A**): TERT expression in cell lines with known and unknown telomere maintenance mechanisms (TMM). (**B**): Magnification of data in A; dotted line represents cut-off value for TERT expression: unknown cell lines with values above this threshold are classified as putative telomerase-positive, those below as putative ALT. (**C**): Telomere length evaluated by TelSeq; putative ALT cell lines with telomere content values below the threshold (shown as a dotted line) are discarded. (**D**): ALT incidence in different types of tumors. (**E**): percentage of types of tumors among all the cell lines analyzed (above) and among the ALT cell lines (below).

TERT expression data from the DepMap portal were used to identify ALT cell lines among those from the CCLE panel for which TMM is unknown (Table S2 in Supplementary Material). The lowest value of TERT expression shown by telomerase-positive cell lines was chosen as the first cut-off (Figure 1A,B). In this way, 65 cell lines showing TERT expression values lower than the cut-off were classified as telomerase-negative and 1105 as telomerase putative (those with values above the cut-off). Subsequently, telomerase-negative cell lines with short telomeres (low telomere content, Figure 1C) were discarded (Table S2 in Supplementary Materials). In this way, 60 cell lines were classified as ALT putative, which, added to 16 cell lines known to be ALT, gives a total of 76.

The use of this stringent method may have led to the exclusion of some cell lines that are ALT-positive and telomerase-negative because they have TERT but no TERC expression. However, the aim of this research was to study the incidence of mutations among ALT cells, and we are quite sure that we have not included telomerase-positive lines among the ones classified as ALT (indeed, while ALT cell lines with TERT expression can exist, no telomerase-positive cell can exist without TERT expression). Moreover, our results show an incidence of ALT of 5.6% (Figure 1D) among CCLE lines, which is similar to previous estimates in tumors [11,12,14]. In particular, the incidence is 23.9% in lines of mesenchymal origin (sarcomas), 6.8% in those of neural origin (which includes also melanomas and neuroendocrine tumors), 3.9% in epithelial lines and 0.9% in blood cell lines (Figure 1D). The fact that mesenchymal cells represent just 8% of all cell lines analyzed but represent 34% of the lines classified as ALT (Figure 1E) confirms the fact that this TMM is more common in sarcomas [12].

In particular, the incidence of ALT is 63% in osteosarcomas (10/16), 25% in chondrosarcomas (1/4), 36% in liposarcomas (4/11), 25% in leiomyosarcomas (1/4), 75% in alveolar rhabdomyosarcoma (6/8), 29% in embryonal rhabdomyosarcomas (2/7), and 0% in Ewing’s sarcomas (0/22). Moreover, we found a 6% incidence in astrocytomas (1/17), 6% in glioblastomas (3/51), 18% in neuroblastomas (3/17), 75% in malignant peripheral nerve sheath tumors (MPNST, 3/4), and 7% in melanomas (6/86).

### 2.2. Incidence of ATRX Alterations and ATRX Expression in ALT Cell Lines

**Figure 2:**
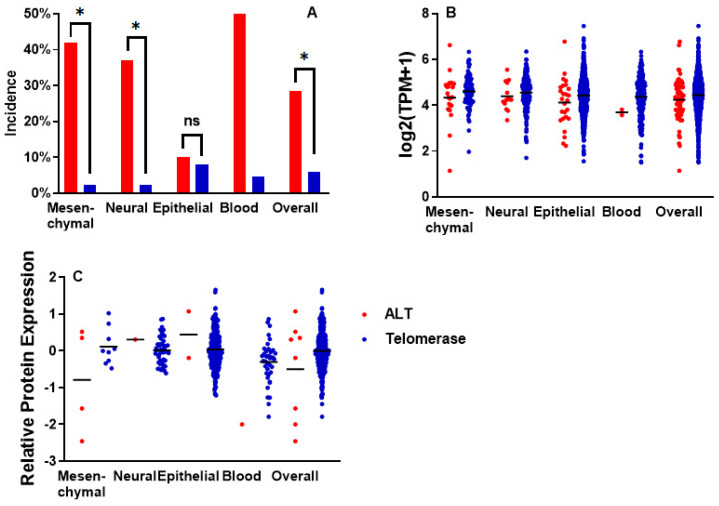
ATRX alterations and expression. (**A**): incidence of ATRX damaging alterations (damaging mutations, copy loss and structural variations; data from 76 ALT-positive and 1299 telomerase-positive cell lines (statistical significance is not shown for blood cell lines, since only 2 are ALT-positive). (**B**): ATRX mRNA expression values (data from 66 ALT-positive and 1235 telomerase-positive cell lines). (**C**): ATRX protein expression values (data from 8 ALT-positive and 342 telomerase-positive cell lines). Asterisks represent significant differences, ns: not significant.

The incidence of *ATRX* alterations (which include damaging mutations, copy number loss, and structural variations, Table S3 in Supplementary Materials) was significantly higher (*p* < 0.0001) in ALT cells compared with telomerase-positive ones (Figure 2A). In particular, this was true for those lines of mesenchymal and neural origin (*p* < 0.0001, both), but not for the epithelial ones (*p* = 0.67). Also, for blood cell lines, the difference was significant (*p* = 0.004), but only 2 of them were ALT lines in this group.

In every group, ATRX RNA expression was lower in ALT cell lines compared with telomerase-positive ones (Figure 2B). The difference was not significant in mesenchymal (*p* = 0.14), neural (*p* = 0.39), epithelial (*p* = 0.06) and blood cell lines (*p* = 0.28). It was significant only in merging all cell lines, independently of their origin (*p* = 0.01).

Data for ATRX protein expression were much scarcer (Figure 2C). Among mesenchymal cell lines, the mean of the 4 ALT cell lines was lower, but not significantly (*p* = 0.11), than the telomerase ones. Among neural cell lines, the only ALT cell line available showed a value higher than the mean of the telomerase ones. Considering all cell lines, independently of their origin, the value for ALT cell lines was lower, but not significantly (*p* = 0.17), than the telomerase ones.

### 2.3. Incidence of Mutations in ALT Cell Lines

**Figure 3:**
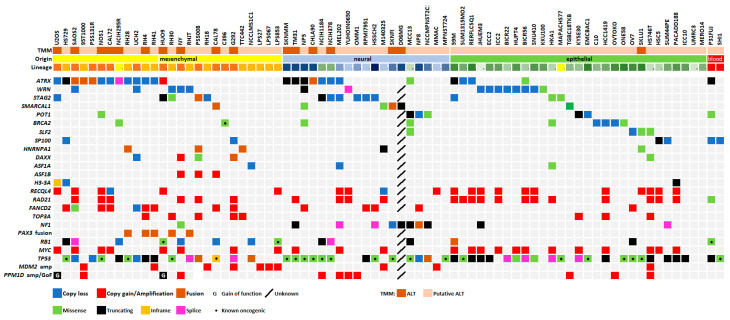
Mutations in ALT cell lines.

Besides *ATRX*, the most common altered genes are *STAG2*, *WRN*, *SMARCAL1*, *POT1*, *BRCA2*, *SLF2*, and *SP100* (Figure 3, Table 1 and Table S3 in Supplementary Material), which all belong to pathways of telomeric integrity maintenance and are known repressors of ALT [15,16,17,18,19,20,21]. Also, *HNRNPA1* showed damaging alterations in some cell lines: this protein allows RPA-to-POT1 switch on telomeric single-stranded DNA at the end of telomere replication [22], and its absence could prolong the presence of RPA (a key enzyme for the first steps of homologous recombination) and thus trigger telomeric recombination [23,24]. Mutations in genes implied in H3 histone deposition (*DAXX*, *ASF1A*, *ASF1B*, *H3-3A*) are much less common (and *ASF1B* showed no mutation at all).

Interestingly, *RAD21*, a cohesion involved in ALT-associated promyelocytic leukemia body [25], and *RECQL4*, a helicase involved in telomeric integrity maintenance [26], are often amplified in ALT cell lines (Figure 3). However, this may merely be due to the fact that they are neighbors of *MYC* (on the chromosome band 8q24), an oncogene often amplified both in ALT and telomerase-positive tumors. We also observed amplifications of *TOP3A*, a gene involved in fork stall resolution [27] and whose amplification has been proposed to be associated with ALT and mutually exclusive with *ATRX* mutations [28] (see below).

Other frequently altered genes are *RB1* and *MYC* (a well-known oncosuppressor and oncogene, respectively), *NF1* (known for being often mutated in neural tumors), and *PAXX* (often fused with *FOXO1* in alveolar rhabdomyosarcomas).

Finally, the p53 pathway is altered in almost all ALT lines (89%), either by *TP53* mutation or *MDM2*, *MDM4*, *PPM1D* amplification, confirming previous observations [29,30]. U2OS cell line (which is *TP53* wild type) has been previously reported as p53 proficient or deficient, depending on the results of *MDM2* copy number evaluation [31,32]. However, Kleiblova et al. [33] finally identified a gain-of-function mutation of *PPM1D* in U2OS that impairs the p53 pathway. We also discovered that the HUO9 cell line harbors this same mutation.

Although it is not one of the main aims of this research, we noted a difference (which may interest the reader) in the types of *TP53* alterations between ALT and telomerase-positive cell lines: whereas the first ones show both point mutations and gene deletions, the latter ones show only point mutations. We explain this with the fact that the *TP53* gene overlaps (partially and antisense) with the *WRAP53* gene (better known as *TCAB1*): being the protein encoded by the latter essential for the assembly of the telomerase enzyme, a loss of this genomic region would be detrimental for telomerase-positive cells but not for ALT ones.

Analysis of mutual exclusivity and co-occurrence of gene alterations gave no significant results. We would mention that unadjusted *p* values were significant for mutual exclusivity between *ATRX* and *POT1* (*p* = 0.048), *ATRX* and *BRCA2* (*p* = 0.048), *WRN* and *STAG2* (*p* = 0.049); however, also in these cases, False Discovery Rate analysis gave no significant results (q = 0.75 each). Since de Nonneville et al. [28] observed mutual exclusivity between *ATRX* mutations and *TOP3A* amplification in osteosarcoma, we tested this hypothesis in our sample. Among the 10 ALT osteosarcoma cell lines, 5 harbored *ATRX* alterations and no *TOP3A* amplification, 2 harbored no *ATRX* alteration and *TOP3A* amplification, and 3 neither of the 2; statistical analysis, however, gave a non-significant result (*p* = 0.44). Looking at TOP3A expression (Figure S1 in Supplementary Material), *ATRX*-wild type, *TOP3A*-amplified cells showed the highest values, and the difference with *ATRX*-deficient, *TOP3A*-wild type ones was significant (*p* = 0.001). However, the difference between *ATRX*-wild type cells (both *TOP3A*-amplified and wild-type) and *ATRX*-deficient, *TOP3A*-wild type ones was not significant (*p* = 0.81). This seems to contradict the hypothesis that in osteosarcoma, either *ATRX* mutations or *TOP3A* overexpression are needed for ALT.

**Figure 4:**
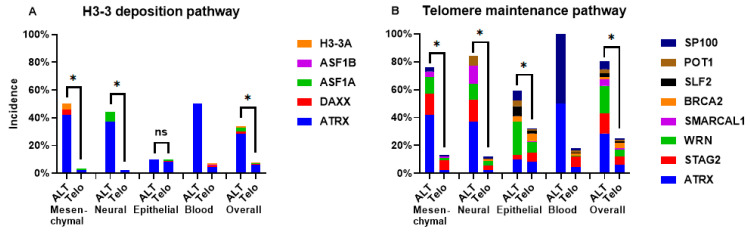
Incidence of damaging alterations of ALT repressor genes. (**A**): genes belonging to the H3.3 histone deposition pathway. (**B**): genes belonging to the telomeric integrity maintenance pathway. Asterisks represent significant differences, ns: not significant (statistical test was not performed on blood cell lines, since only 2 are ALT-positive). Incidence values are calculated from 76 ALT-positive and 1299 telomerase-positive cell lines.

We previously confirmed that *ATRX* is the most frequently altered gene in ALT cells (Figure 3). Its alteration can be viewed as a defect in the H3-3 histone deposition pathway, leading to telomeric euchromatinization and thus ALT establishment, as proposed in the past [8]. However, other members of this pathway (*DAXX*, *ASF1A*, *ASF1B*, *H3-3A*) are rarely altered in our ALT cell line panel (Figure 3). In particular, the percentage of cell lines with one of these genes being altered is significantly higher in ALT compared with telomerase-positive cells (Figure 4A), if we consider mesenchymal and neural lines (*p* < 0.0001, both), but not in epithelial ones (*p* = 0.46).

ATRX absence, on the other hand, can be viewed as the cause of defects in telomeric fork resolution, cohesion, and unwinding (which, also in this case, can lead to ALT). The percentage of cell lines with alterations in genes belonging to this pathway (Figure 4B) is significantly higher in ALT cells compared with telomerase-positive ones; this is true for mesenchymal, neural, and also epithelial lines (*p* < 0.0001, for all).

## 3. Discussion

Since ALT is much more common in mesenchymal and neural tumors [11,12], research on the role of ATRX has been conducted almost solely in these tumor types. In this study, we identified a wider panel of ALT-positive cell lines, including carcinomas, and we estimated the incidence of *ATRX*-damaging mutations among them. Our method may have given some false negatives (i.e., we have excluded some true ALT-positive cell lines), but we are sure to have not included false negatives (i.e., ALT-negative identified as ALT-positive). When we began this study, the TMMs of RH4, HS729, and TM31 were unknown, and we, using the protocol presented above, predicted that they were ALT-positive (Figure 3). Before we finished writing this article, we experimentally demonstrated that RH4 was ALT-positive [34]. Moreover, HS729 and TM31 were confirmed as ALT positive [35] (M. Zimmermann personal communication), giving us confidence in the robustness of our method. Also, the similarity between our results and the incidence of ALT in tumor samples [11,12,14] is encouraging.

Data on ATRX mRNA expression seemed to show a trend for lower values in ALT cell lines, but this is not statistically significant. Moreover, many ATRX-deficient ALT cell lines showed high values. This is because these cell lines may transcribe mRNA from this gene, although this mRNA will not be translated into a functional protein. Therefore, protein expression data would be much more informative for the investigation of ATRX presence in the different cell lines. Unfortunately, protein expression data are very scarce (Figure 2). Therefore, the best way to study the ATRX status of the different cell lines is to investigate the presence of gene mutations and alterations. We have shown that *ATRX* is the most altered gene in ALT cell lines with a mesenchymal or neural origin but not among those with an epithelial one (Figure 2A and Figure 3).

The first (and still popular) hypothesis linking ATRX loss and ALT is that without ATRX-mediated deposition of H3.3 histone, telomeres become euchromatic and prone to recombination [8]. It should be added, however, that the actual euchromatic/heterochromatic status of telomeres is still not clear [36]. Following this hypothesis, replication stress at telomeres would be a consequence, or by-product, of the absence of ATRX and its helicase activity. Other authors [37], instead, consider the telomeric replication stress (caused by ATRX inactivation) as the cause of ALT: this stress, in fact, consists of the stall and collapse of the replication fork, which activates HR at telomeres. If ALT is triggered by loss of telomeric H3.3 histone, we should find mutations not only in *ATRX* but also in other genes involved in this pathway. Our results, however, show that these (including mutations of *H3-3A*) are very rare among ALT-positive cell lines (Figure 4A). It could be objected that this may be due to the lethality of this kind of mutation. However, they are present in other types of tumors and, in particular, *H3-3A* mutations have a high incidence among giant cell tumors of bone [38], and so far, all the tumors of this type analyzed have active telomerase [39]. On the other hand, the incidence of loss of proteins involved in fork stall resolving (SMARCAL1, WRN, SLF2, BRCA2) and repression of recombination (STAG2, POT1, SP100, Figure 4B) makes us believe that ALT is triggered by replication stress at telomeres rather than the absence of H3.3 deposition. For clarity, we must add that knocking down of SMARCAL1 [21], WRN [40], SLF2 [21], BRCA2 [19], STAG2 [18], POT1 [16], and SP100 [15] have been shown to induce activation of ALT or, at least, of some of its markers (C-Circles, telomeric recombination, etc.).

However, even if one disagrees with us (and thus believes that ATRX loss triggers ALT because H3.3 deposition is hampered), it should be noted that ATRX loss is peculiar to mesenchymal and neural ALT cell lines and almost absent in epithelial ones; thus, loss of H3.3 deposition would be a mechanism for ALT establishment only in mesenchymal and neural cells and not a universal feature.

Also, in vitro, ATRX loss increases the proportion of cell lines activating ALT in p53-defective fibroblasts but not in p53-defective epithelial cells [41].

From our results, it seems that for ALT establishment, a defect in the telomeric fork repair pathway is needed and that *ATRX* alterations are specifically enriched in mesenchymal and neural ALT cell lines. This could be due to other functions exerted by ATRX specifically in mesenchymal and neural (but not in epithelial) cells that need to be hampered. In addition to its telomeric role, ATRX is also involved in regulating the expression of imprinted genes [42]. ATRX protein is highly enriched at all known imprinting control regions [43,44] and in the absence of Atrx, H19 and Igf2 expression are increased in mouse brain [43,44,45]. Atrx-null mice also show the downregulation of Neuroligin 4 and other enzymes important for neuronal differentiation [45], and, in general, ATRX loss impairs neurodifferentiation [46]. Although the nervous system has attracted most of the attention, some studies investigated the effects of ATRX inactivation on muscle cells and osteoblasts, showing an inability to differentiate [47,48]. Thus, ATRX loss seems to induce replication stress and impaired differentiation in neural and mesenchymal cells, giving at once two steps in the process of ALT tumorigenesis. Is ATRX inactivation dispensable for epithelial differentiation? Answering this question would help elucidate differences in ATRX loss incidence between ALT epithelial and mesenchymal/neural cells. Similar hypotheses can be also drawn for SMARCAL1 (which is often lost in ALT glioblastomas and is mutually exclusive with ATRX). In fact, besides its well-known role as a helicase responsible for telomeric integrity maintenance, it has recently been shown that its loss impairs differentiation [49].

In addition to altering the expression of genes driving differentiation, ATRX loss (as said before) impairs the expression of imprinted genes and, for example, increases the levels of H19 and IGF2 [45]. IGF2 is a growth factor [50], and H19 is a non-coding RNA essential for tumorigenesis [51] that is reactivated in most tumors [52]. Moreover, H19 is upregulated by oncogenes, such as c-Myc [52]. One study showed that H19 and IGF2 are downregulated during fibroblast senescence and that reactivation of H19 upregulates IGF2, increasing proliferation and avoiding senescence [53]. On the other hand, in prostate epithelial and urothelial cells, aging is associated with loss of imprinting of IGF2, leading to increased expression [54]. Thus, it is tempting to put forward this hypothesis: (a) ALT cells (which usually lack alterations in genes such as *Myc*) need reactivation of factors like IGF2 or H19 in order to bypass senescence and/or differentiation; (b) ATRX loss is needed in mesenchymal and neural cells in order to reactivate these imprinted genes; (c) ATRX loss is not needed in epithelial cells because they reactivate IGF2 during senescence. Of course, there are few studies to support this speculation, but we think that this issue (i.e., loss of imprinting due to ATRX mutations in different tissues) deserves further research.

**Figure 5:**
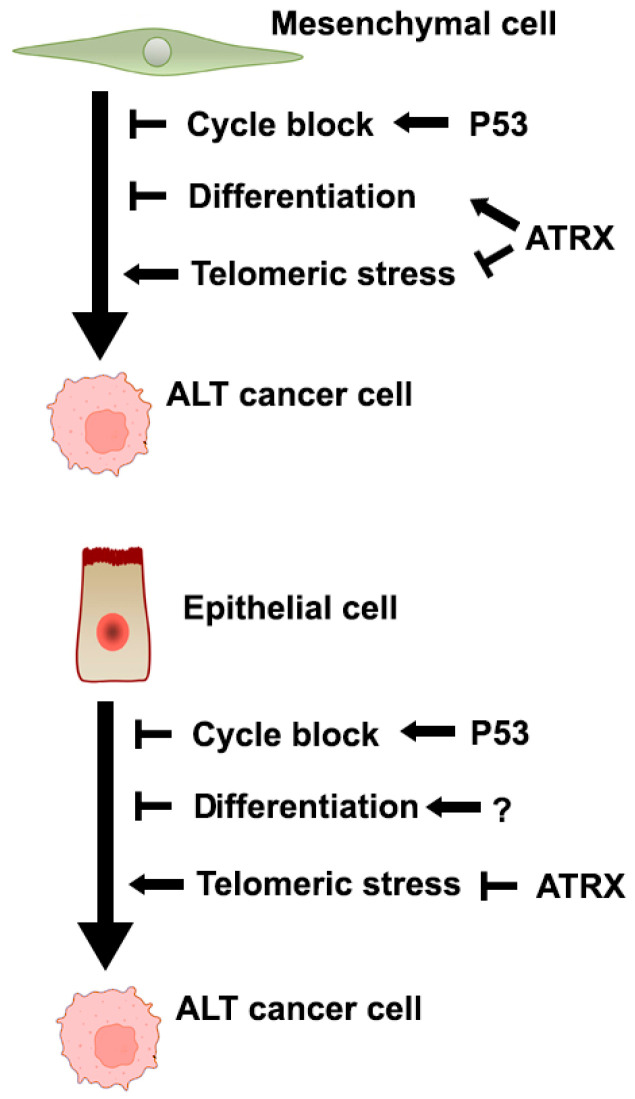
Hypothesis on the different roles of ATRX in mesenchymal and epithelial cells. Small arrows (←) indicate stimulation; blunt arrows (⊢) indicate inhibition.

In conclusion, viewing ALT establishment as a “multi-hit” process, we hypothesized the need for inactivation of the p53 pathway (in order to cope with replication and telomeric stress [55,56]), of the “telomeric integrity maintenance” pathway (causing de-repression of telomeric recombination) and of other ones (yet to be discovered) that may be lineage specific. As in mesenchymal and neural cells, one of these unknown pathways may rely on ATRX, the inactivation of this gene “kills two birds with one stone” (de-repression of telomeric recombination and lineage-specific pathway) in these lineages but gives only one “hit” in epithelial cells (de-repression of telomeric recombination), explaining the higher incidence of ATRX inactivation in the former (Figure 5). As we have helped to identify more ALT epithelial cell lines, our last recommendation is to perform more studies on these in order to have a clearer understanding of the ALT phenomenon.

## 4. Material and Methods

Cell lines present in the DepMap portal (https://depmap.org/portal, accessed on 9 July 2025), version 2023Q2, were classified, using literature data, according to their TMM, i.e., as ALT, or telomerase-positive, or without TMM. Those with no data in the literature were classified as unknown. The criterion for classifying a cell line as telomerase-positive was the presence of measured telomerase activity in the literature. The criterion for classifying a cell line as ALT was the absence of telomerase activity and the presence of ALT markers (long and heterogeneous telomeres, C-circles, ALT-associated PML Bodies). Three cell lines are reported in the literature as cancerous but with no TMM (i.e., telomerase- and ALT-negative). Thirty-eight cell lines are reported as fibroblasts in the DepMap portal; these were used just for comparison of TERT expression and telomere content values. Cell lines classified as unknown were subsequently subdivided on the basis of their TERT expression values (downloaded from the DepMap portal): the lowest value of TERT expression shown by known telomerase-positive cell lines was chosen as a threshold, and those cell lines with values above it were classified as “telomerase putative”. Vice versa, cell lines with values below this threshold were classified as “telomerase-negative”. The latter were further scrutinized for telomere length using data of “telomere content” obtained with TelSeq [57] and present in the DepMap portal [58]. Those cell lines with telomere content value below −1 (corresponding to 5 kb) were interpreted as having short telomeres and thus discarded. “Telomerase-negative” cell lines with telomere content value above −1 were classified as “ALT putative”.

ATRX RNA and protein expression and mutation data were downloaded from the DepMap portal. A gene was considered to have a damaging alteration if it had one or more of the following: nonsense mutation, out-of-frame insertion or deletion, splice site mutation, fusion, or copy number loss. Missense mutations were not considered as damaging unless they are reported as oncogenic in the literature or databanks.

Comparison of (log-transformed) expression data was performed using Student’s *t* test. Comparison of incidence data was performed using the Chi-squared test. In order to test mutual exclusivity or co-occurrence of gene alterations, we used a one-sided Fisher’s Exact Test. We also used the Log2 Odds Ratio to assess the direction of association, where negative values indicate trends towards mutual exclusivity and positive values indicate trends toward co-occurrence. To adjust for multiple hypotheses across multiple gene pairs, we used the Benjamini–Hochberg False Discovery Rate correction procedure.

## Figures and Tables

**Table 1:** Most frequent alterations in ALT cell lines. Note that *ATRX* belongs both to the telomeric integrity and the H3-3 deposition pathway.

Pathway	Genes	Alterations
	*ATRX*	Frequent damaging alterations in mesenchymal (42%) and neural cells (37%), much rarer in epithelial ones (10%)
Telomeric integrity	*WRN*	Damaging alterations in epithelial (24%), mesenchymal (12%) and neural cells (11%)
	*STAG2*	Damaging alterations in mesenchymal (15%) and neural cells (16%), much rarer in epithelial ones (3%)
	*SMARCAL1*	Damaging alterations in neural cells (13%), much rarer in mesenchymal cells (4%), absent in epithelial ones
H3-3 deposition	*DAXX*, *ASF1A*, *H3-3A*	Quite rare damage mutations in mesenchymal and neural cells (<7%), none in epithelial ones
	*TOP3A*	Amplified in 11% of cell lines
Cell proliferation	*RB1*	Damaging alterations in 19% of cell lines
	*MYC*	Amplified in 31% of cell lines
P53	*TP53*	Frequent damaging alterations (68%)
	*MDM2*	Amplified in 13% of cell lines

## Data Availability

Original data were retrieved from the DepMap portal (https://depmap.org/portal, accessed on 9 July 2025). All data used in this article are available in the Supplementary Material. This paper does not report original code.

## Supplementary Materials

The following supporting information can be downloaded at: https://www.mdpi.com/article/10.3390/ijms26146765/s1. References [59,60,61,62,63,64,65,66,67,68,69,70,71,72,73,74,75,76,77,78,79,80,81,82,83,84,85,86,87,88,89,90,91,92,93,94,95,96,97,98] are cited in the supplementary materials.
