# Supplementary material for: Alternative Lengthening of Telomeres: The Need for ATRX Mutations Is Lineage-Dependent
Source: Int J Mol Sci. 2025 Jul 15;26(14):6765. doi: 10.3390/ijms26146765 (PMC12295417; doi:10.3390/ijms26146765)
Supplement: Supplementary file 1 [file ijms-26-06765-s001.zip › ijms-3697762_Supplementary.pdf]

Alternative Lengthening of Telomeres: the need for *ATRX* mutations is lineage-dependent – Supplementary material

Ion Udroiu, Antonella Sgura

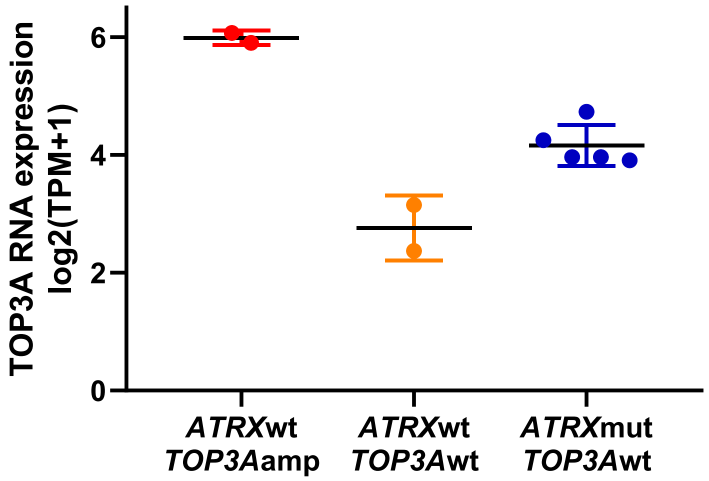

Figure S1. TOP3A expression in ALT osteosarcoma cell lines. Difference between *ATRX*-wild type, *TOP3A*-amplificated cells and *ATRX*-deficient, *TOP3A*-wild type ones was significant (p=0.001). Difference between *ATRX*-wild type cells (both *TOP3A*-amplificated and wild-type) and *ATRX*-deficient, *TOP3A*-wild type ones was not significant (p=0.81).
